# The critical relationship between vaginal microecology and *Ureaplasma urealyticum:* a retrospective study

**DOI:** 10.7717/peerj.19783

**Published:** 2026-01-05

**Authors:** YanHong Liu, Jie Zheng, Junpeng Zhao, Yuhong Yao, Dongxue Gao, Wenjie Qi, Yingmei Wang, Jinyin Yan

**Affiliations:** 1 Clinical Laboratory, Tangshan Fengnan District Hospital, Tangshan, Hebei, China; 2 Centers for Disease Control and Prevention, Tangshan, Hebei, China; 3 Food and Drug Inspection Center, Tangshan, Hebei, China; 4 Department of Reproductive Genetics, Tangshan Maternal and Children Health Hospital, Tangshan, Hebei, China; 5 Nursing Department, Tangshan Workers’ Hospital, Tangshan, Hebei, China; 6 Department of Obstetrics and Gynecology, Tangshan Maternal and Children Health Hospital, Tangshan, Hebei, China; 7 Nursing Department, Tangshan Fengnan District Hospital, Tanagshan, Hebei, China; 8 Department of Breast Surgery, Tangshan Central Hospital, Tangshan, Hebei, China

**Keywords:** Ureaplasma urealyticum, Vaginal microecology, Infection, Association

## Abstract

**Background:**

Vaginal microecology can reveal the health of the female reproductive tract directly. Female vaginal microecology reflects the state of female reproductive tract health. This study aimed to utilize a variety of female vaginal microecological indicators to comprehensively assess the relationship between the level of vaginal microecological health and *Ureaplasma urealyticum* (UU) infection in women.

**Methods:**

A total of 408 participants were included in this study, including 144 UU-positive and 264 UU-negative individuals. Clinical information of the participants was collected, and vaginal microecological indicators (cleanliness, hydrogen peroxide (H_2_O_2_), leukocyte esterase (LEU), sialidase (SNA), N-acetyl glucosidase (NAG), and *β*-glucuronidase (GUS)) were tested. The measurement data were expressed as mean ± standard deviation (x ± s), and the comparison of data between groups was performed using a *t*-test; count data were expressed as the number of cases (percentage) (*n*[%]), and the data between groups were compared using the chi-square test. Univariate and multivariate logistic regression model analyses explored the factors modifying infection with UU.

**Results:**

UU-positive patients exhibited higher rates of cleanliness positivity, H_2_O_2_ positivity, LEU positivity, SNA positivity, NAG positivity, and GUS compared to UU negative patients (*P* < 0.05) . The univariate logistic regression model found that cleanliness, H_2_O_2_, LEU, SNA, NAG, and GUS were risk factors for UU infection in women (Cleanliness: odds ratio [*OR*] = 4.30, 95% confidence interval [*CI*] [2.79–6.63]); H_2_O_2_: *OR* = 9.01, 95% *CI* [5.33–15.23]; LEU: *OR* = 1.88, 95% *CI* [1.22–2.91]; s SNA: *OR* = 5.53, 95% *CI* [2.73–11.19]; NAG: *OR* = 2.41, 95% *CI* [1.35–4.30]; and GUS: *OR* = 1.95, 95% *CI* [1.21–3.15]) . The multivariate logistic regression model found that the independent risk factors for UU infection in patients were cleanliness (*OR* = 3.00, 95% *CI* [1.66–5.43]) and H_2_O_2_ (*OR* = 7.24, 95% *CI* [4.19–12.51]).

**Conclusions:**

Vaginal cleanliness and H_2_O_2_ abnormalities are risk factors for UU infections in women. Therefore, female UU infections can be prevented by maintaining vaginal microecology.

## Introduction

*Ureaplasma urealyticum* (UU) is a bacterium included in the genus *Ureaplasma*. UU is a commonly found microorganism in the human reproductive tract and is generally associated with reproductive tract infections. Infection with UU manifests itself clinically as an extensive range of symptoms, particularly in women, and may lead to diseases such as vaginitis and pelvic inflammatory disease (Bender & Gundogdu, 2022; Liu et al., 2023). The proportion of UU infections varies in the bacterial vaginosis (BV) and aerobic vaginitis (AV) groups compared to healthy women (Rumyantseva et al., 2018). Thus, vaginal dysbiosis and different flora status reflect different vaginosis in women (Abou Chacra, Fenollar & Diop, 2022). Detection of interleukin-1beta (IL-1beta) and IL-1 receptor antagonist (IL-1ra) levels in vaginal specimens from pregnant women in early pregnancy has been associated with UU infection (Doh et al., 2004). However, UU positivity in the female vagina is not associated with abnormal vaginal secretions or pH (Plummer et al., 2021). The female vaginal microecology is a complex and dynamic microecosystem composed of vaginal microorganisms, the endocrine system, and the local immune system, and normal vaginal microbiota is essential for maintaining microecosystem homeostasis. Therefore, the relationship between patients with UU infections and vaginal microecological health status remains to be determined. In the future, to prevent and treat vaginal diseases more effectively, it will be necessary to explore and the impact of UU on the microecology of the female vagina.

**Figure 1:**
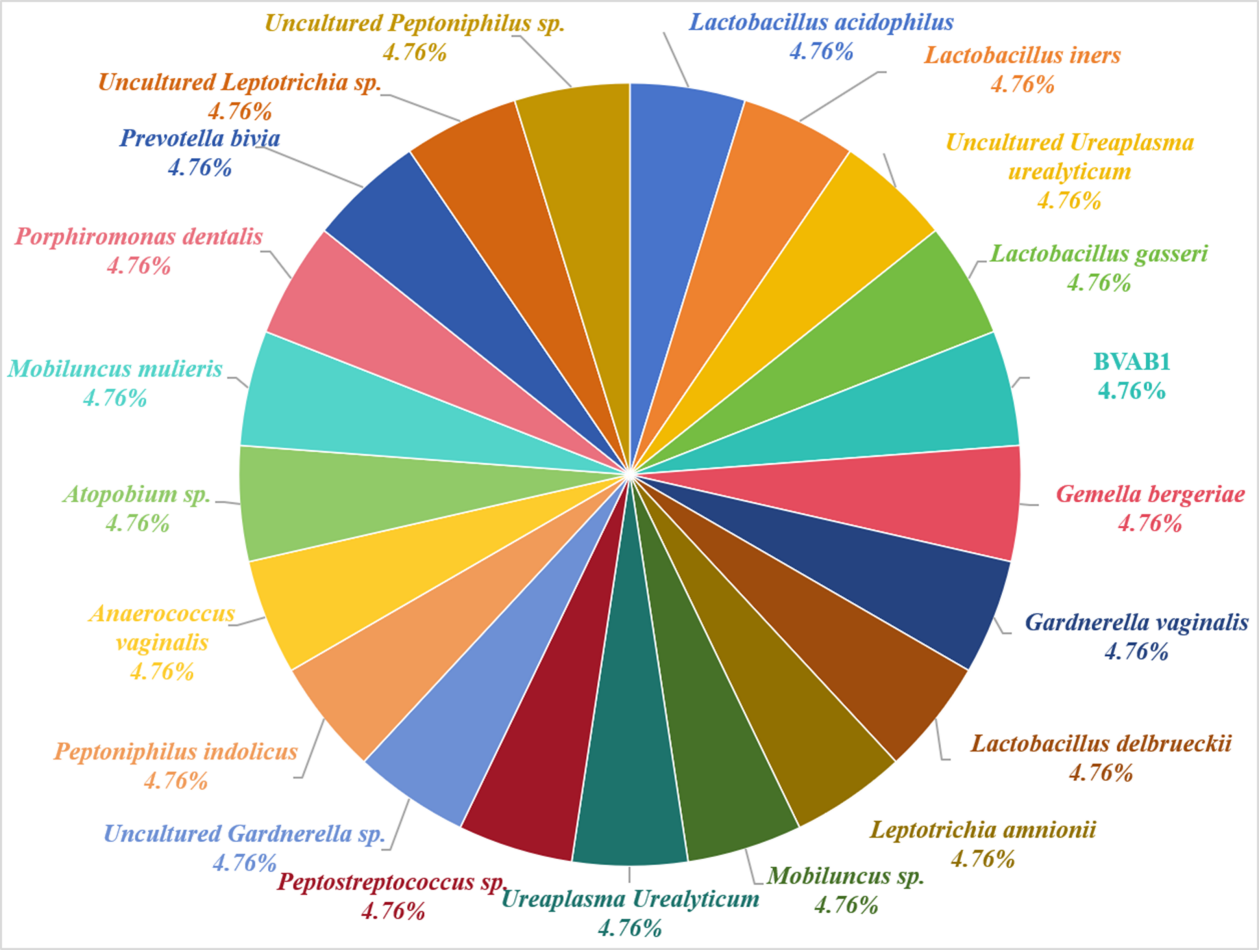
Microorganisms in the vagina of women.

The female vagina is a complex microbial ecosystem. The female reproductive tract’s microbiome reflects the woman’s health, and a healthy microbiome consists mainly of *Lactobacillus* (Moreno & Simon, 2018). Hernández-Rodríguez et al. (2011) tested vaginal secretions from healthy pregnant women and identified 21 different microorganisms, with the highest positive detection rate of 21% for the UU, which is associated with bacterial vaginosis (Fig. 1). The women’s vaginal microecology changes continuously before, during, and after childbirth during puberty, menstruation, pregnancy, and menopause (Shen et al., 2022). Human vaginal microecology imbalances can lead to inappropriate inflammatory responses and abnormal immune responses, leading to a wide range of female reproductive health challenges (Shen et al., 2022). The levels of vaginal hydrogen peroxide (H_2_O_2_) are associated with the presence of a wide range of microorganisms (Hillier et al., 1993). Several studies have indicated that six indicators such as pH, H_2_O_2_, leukocyte esterase (LEU), sialidase (SNA), *β*-glucuronidase (GUS), and acetylglucosidase assist in understanding vaginal microecology and diagnosing vaginitis and cervical cancer (Feng et al., 2024; Li et al., 2020). Additionally, Wang, Wan & Zhang (2024) found that Systemic Immune Inflammation Index (SII) is associated with Mycoplasma pneumonia, and can be used as a valid indicator for predicting its severity in children. Patients with UU infection are associated with abnormalities in inflammatory markers such as tumor necrosis factor (TNF)-alpha, interleukin-6, and toll-like receptor 2 (Jacobsson et al., 2009; Hassanein et al., 2012; Noh et al., 2019). Therefore, vaginal microecology may assist in better understanding, studying, and treating UU. Studies have indicated that vaginal microecology may be associated with human papillomavirus, vaginitis, and other female reproductive tract disease infections (Ilhan et al., 2019; Fan et al., 2024; Wei et al., 2022). Always although both vaginal microecology and UU positivity are associated with female diseases, the relationship between them has not been clarified.

The monitoring of vaginal microecology-related and inflammation indicators can assist in the diagnosis and adjuvant treatment of vagina-related diseases in women. Therefore, this study aimed to explore the association between vaginal microecological factors and UU infection to provide new ideas for further research and clinical treatment of UU.

## Materials & Methods

### General information

Female participants who attended the outpatient clinic of the Department of Obstetrics and Gynaecology at Tangshan Workers Hospital and Tangshan Fengnan District Hospital from June 2021 to June 2024 were included in this retrospective study. Participants who had received antibiotic treatment in the last month, those with other vaginal pathogenic bacteria infections, and those with missing samples or insufficient sample size were excluded. A total of 408 study participants were finally included, including 144 UU-positive and 264 UU-negative individuals. The study was reviewed and approved by the Clinical Research Ethics Committee of Tangshan Fengnan District Hospital (approval number: 2022-05). Each participants provided written informed consent after receiving a detailed explanation of the study purposes and study procedures.

### Sample collection

A doctor collected vaginal samples for UU testing (Feng et al., 2024). At the time of the speculum examination of the study subjects, the physician used two sterile disposable swabs to collect vaginal secretions from the subject’s posterior vaginal fornix. One sample was stored in a refrigerator at 2–4 °C and had to be completed on the day of sampling for vaginal microbiology and vaginal cleanliness testing. The other sample was stored at −80 °C for UU testing.

Approximately three mL of peripheral blood of the participants was collected to detect SII.

### Sample testing

This study used real-time fluorescent quantitative polymerase chain reaction (PCR) to detect UU in the samples. *Ureaplasma urealyticum* Fluorescent Polymerase Chain Reaction Diagnostic Kit (Daan Gene Co., Ltd., Guangzhou, China) was used in this study. Concentrations of U. urealyticum DNA ≥500 copies/ml were considered positive (Yoshida et al., 2022).

The vaginal microecological balance of the participants was observed by the Vaginitis Diagnostic kit (Zhuhai Lituo Biotechnology Co., Ltd, Zhuhai China), which included H_2_O_2_ (H_2_O_2_ results were determined as H_2_O_2_ (negative) when H_2_O_2_ ≥2 µmol/L in vaginal secretions, indicating normal microbial function in the vagina; otherwise, the results were positive), LEU, SNA, N-acetyl glucosidase (NAG), GUS (Feng et al., 2024).

Vaginal cleanliness (Zheng et al., 2019a; Zheng et al., 2019b): Vaginal discharge was observed microscopically and vaginal cleanliness was categorized as I. to IV based on the number of bacilli, epithelial cells and white blood cells (WBC). Grade I–II indicates normal vaginal cleanliness, and grade III–IV indicates abnormal vaginal cleanliness. Grade I is the presence of a large number of Lactobacillus vaginalis, epithelial cells, and no other bacteria in the vaginal discharge observed under the microscope, with a white blood cell count of 0–5/HP. Grade II is the presence of some Lactobacillus and vaginal epithelial cells, some pus cells, and other bacteria observed microscopically in vaginal discharge with a WBC of 10–15/HP. Grade III is the presence of a few Lactobacillus, pus cells, and other bacteria observed microscopically with a WBC of 15–30/HP. Grade IV is the presence of no Lactobacillus but pus cells and other bacteria observed microscopically with a WBC of more than 30/HP. Grade V is the absence of Lactobacillus, but pus cells and other bacteria were observed microscopically with a WBC of more than 30/HP.

Neutrophil, lymphocyte, and platelet counts were measured using a BC-3200 automatic blood cell analyzer. SII was calculated, SII = (platelet count × neutrophils count)/lymphocytes count.

### Statistical analysis

The data of participants were analyzed using SPSS23.0 software. Among them, the measurement data were expressed as mean ±standard deviation (x ±s). Moreover, the *t*-test was used for the data between groups; the count data were expressed as cases (percentage) (*n* [%]), and the chi-square test was used. The factors influencing infection with UU were explored through univariate and multivariate logistic regression model analyses. The test level was set at *α* = 0.05, and differences were considered statistically significant at *P* < 0.05.

## Results

### Study population

A total of 408 female participants, including 144 UU-positive and 264 UU-negative, were included. As illustrated in Table 1, the percentage of UU-positive participants with high vaginal cleanliness (34.03%) was lower than the UU-negative population (68.94%, *P* <  0.05); the percentage of UU-positive participants with vaginal H_2_O_2_ positivity (85.42%) was higher than the UU-negative population (39.39%, *P* <  0.05); the percentage of UU-positive participants with LEU-positive participants (71.53%) accounted for a higher proportion than the UU-negative population (57.20%, *P* <  0.05); SNA-positive participants (20.83%) accounted for a higher proportion of UU-positive participants than the UU-negative population (4.55%, *P* <  0.05); NAG-positive participants (20.14%) was higher than the UU-negative population (9.47%, *P* < 0.05); and the percentage of UU-positive participants with GUS positivity (29.17%) was higher than the UU-negative population (17.42%, *P* < 0.05). The comparison of data in the age, age of menarche, number of pregnancies, number of miscarriages, and SII groups between the two groups of participants demonstrated no statistically significant difference (*P* > 0.05).

**Table 1:** The intergroup comparison of clinicopathological characteristics ([x±s], *n* [%]).

Characteristics		All (*n* = 408)	UU-Negative (*n* = 264)	UU-Positive (*n* = 144)	*x* ^2^ */t*	*P*-value
Age (year)		36.48 ± 11.55	36.11 ± 12.07	37.15 ± 10.56	−0.87	0.383
Age of menarche (year)		13.51 ± 1.11	13.51 ± 1.04	13.52 ± 1.23	−0.08	0.938
Number of pregnancies	<=1	104 (25.49)	65 (24.62)	39 (27.08)	0.30	0.586
	>1	304 (74.51)	199 (75.38)	105 (72.92)		
Number of miscarriages	<=1	171 (41.91)	108 (40.91)	63 (43.75)	0.31	0.578
	>1	237 (58.09)	156 (59.09)	81 (56.25)		
SII	Low	204 (50.00)	136 (51.52)	68 (47.22)	0.69	0.407
	High	204 (50.00)	128 (48.48)	76 (52.78)		
cleanliness	I or II	231 (56.62)	182 (68.94)	49 (34.03)	46.24	<0.001
	III or IV	177 (43.38)	82 (31.06)	95 (65.97)		
H_2_O_2_	Negative	181 (44.36)	160 (60.61)	21 (14.58)	79.96	<0.001
	Positive	227 (55.64)	104 (39.39)	123 (85.42)		
LEU	Negative	154 (37.75)	113 (42.80)	41 (28.47)	8.14	<0.001
	Positive	254 (62.25)	151 (57.20)	103 (71.53)		
SNA	Negative	366 (89.71)	252 (95.45)	114 (79.17)	26.77	<0.001
	Positive	42 (10.29)	12 (4.55)	30 (20.83)		
NAG	Negative	354 (86.76)	239 (90.53)	115 (79.86)	9.24	0.002
	Positive	54 (13.24)	25 (9.47)	29 (20.14)		
GUS	Negative	320 (78.43)	218 (82.58)	102 (70.83)	7.59	0.006
	Positive	88 (21.57)	46 (17.42)	42 (29.17)		

**Notes.**

H_2_O_2_ Hydrogen peroxide LEU Leukocyte esterase SNA sialidase NAG N-acetyl glucosidase GUS *β*-glucuronidase *P*-value probability value

### Univariate logistic analysis of the association between vaginal microecology and UU

Univariate logistic regression analyses of participants basic clinical information, SII, and vaginal microecology were conducted to find risk factors for UU infection in women. As shown in Table 2, participants with low cleanliness (III or IV) had a significantly higher risk of UU infection than that in those with high cleanliness (I or II) (odds ratio [*OR* ] = 4.30, 95% confidence interval [*CI* ] [2.79–6.63], *P*  < 0.001); H_2_O_2_-positive participants had a significantly higher risk of UU infection compared to H_2_O_2_-negative participants (*OR* = 9.01, 95% *CI* [5.33–15.23], *P* < 0.001); LEU-positive participants had a significantly higher risk of UU infection compared to LEU-negative participants (*OR* = 1.88, 95% *CI* [1.22–2.91], *P* < 0.001); SNA-positive participants had a significantly higher risk of UU infection compared to SNA-negative participants (*OR* = 5.53, 95% *CI* [2.73–11.19], *P* <  0.001); NAG-positive participants had a significantly higher risk of UU infection than NAG-negative participants (*OR* = 2.41, 95% *CI* [1.35 to −4.30], *P* = 0.003); GUS-positive participants had a significantly higher risk of UU infection than GUS-negative participants (*OR* = 1.95, 95% *CI* [1.21–3.15], *P* = 0.006). However, no significant association was observed between age, age of menarche, number of pregnancies, number of miscarriages, and SII and infection with UU in women (*P* > 0.05).

**Table 2:** Univariate logistic analysis of the association between the vaginal microecology and *Ureaplasma urealyticum* (UU) (*n*[%]).

Characteristics		All (*n* = 408)	UU-Negative (*n* = 264)	UU-Positive (*n* = 144)	*OR* (95% *CI*)	*P*-value
Age (year)		36.48 ± 11.55	36.11 ± 12.07	37.15 ± 10.56	1.01 (0.99–1.03)	0.382
Age of menarche (year)		13.51 ± 1.11	13.51 ± 1.04	13.52 ± 1.23	1.01 (0.84–1.21)	0.934
Number of pregnancies	<=1	104 (25.49)	65 (24.62)	39 (27.08)	Reference	Reference
	>1	304 (74.51)	199 (75.38)	105 (72.92)	0.88 (0.55–1.40)	0.586
Number of miscarriages	<=1	171 (41.91)	108 (40.91)	63 (43.75)	Reference	Reference
	>1	237 (58.09)	156 (59.09)	81 (56.25)	0.89 (0.59–1.34)	0.578
SII	Low	204 (50.00)	136 (51.52)	68 (47.22)	Reference	Reference
	High	204 (50.00)	128 (48.48)	76 (52.78)	1.19 (0.79–1.78)	0.407
cleanliness	I or II	231 (56.62)	182 (68.94)	49 (34.03)	Reference	Reference
	III or IV	177 (43.38)	82 (31.06)	95 (65.97)	4.30 (2.79–6.63)	<0.001
H_2_O_2_	Negative	181 (44.36)	160 (60.61)	21 (14.58)	Reference	Reference
	Positive	227 (55.64)	104 (39.39)	123 (85.42)	9.01 (5.33–15.23)	<0.001
LEU	Negative	154 (37.75)	113 (42.80)	41 (28.47)	Reference	Reference
	Positive	254 (62.25)	151 (57.20)	103 (71.53)	1.88 (1.22–2.91)	<0.001
SNA	Negative	366 (89.71)	252 (95.45)	114 (79.17)	Reference	Reference
	Positive	42 (10.29)	12 (4.55)	30 (20.83)	5.53 (2.73–11.19)	<0.001
NAG	Negative	354 (86.76)	239 (90.53)	115 (79.86)	Reference	Reference
	Positive	54 (13.24)	25 (9.47)	29 (20.14)	2.41 (1.35–4.30)	0.003
GUS	Negative	320 (78.43)	218 (82.58)	102 (70.83)	Reference	Reference
	Positive	88 (21.57)	46 (17.42)	42 (29.17)	1.95 (1.21–3.15)	0.006

**Notes.**

H_2_O_2_ Hydrogen peroxide LEU Leukocyte esterase SNA sialidase NAG N-acetyl glucosidase GUS *β*-glucuronidase *P*-value probability value *CI* confidence interval *OR* odds ratio

### Multivariate logistic analysis of the association between vaginal microecology and UU

To explore the independent risk factors for UU infection risk, statistically significant variables (*P* <  0.05) in the univariate logistic regression analysis in the subsequent multivariate logistic regression analyses were included (Table 3). The endpoint dependent variable was whether the patient was infected with UU (negative = 0, positive = 1) and multivariate logistic analysis was performed with cleanliness (negative = 0, positive = 1), H_2_O_2_ (negative = 0, positive = 1), LEU (negative = 0, positive = 1), SNA (negative = 0, positive = 1), NAG (negative = 0, positive = 1), and GUS (negative = 0, positive = 1) as independent variables for multivariate logistic analysis. Cleanliness (*OR* = 3.00, 95% *CI* [1.66–5.43], *P* < 0.001) and H_2_O_2_ (*OR* = 7.24, 95% *CI* [4.19–12.51], *P* < 0.001) were independent risk factors for UU infection in participants. However, the remaining vaginal microecological factors (LEU, SNA, NAG, GUS) have not shown an independent risk of UU infection (*P* > 0.05).

**Table 3:** Multivariate logistic analysis of the association between the vaginal microecology and *Ureaplasma urealyticum* (UU) (n [%]).

Characteristics		All (*n* = 408)	UU-Negative (*n* = 264)	UU-Positive (*n* = 144)	*OR* (95% *CI*)	*P*-value
cleanliness	I or II	231 (56.62)	182 (68.94)	49 (34.03)	Reference	Reference
	III or IV	177 (43.38)	82 (31.06)	95 (65.97)	3.00 (1.66–5.43)	<0.001
H_2_O_2_	Negative	181 (44.36)	160 (60.61)	21 (14.58)	Reference	Reference
	Positive	227 (55.64)	104 (39.39)	123 (85.42)	7.24 (4.19–12.51)	<0.001
LEU	Negative	154 (37.75)	113 (42.80)	41 (28.47)	Reference	Reference
	Positive	254 (62.25)	151 (57.20)	103 (71.53)	0.86 (0.48–1.53)	0.611
SNA	Negative	366 (89.71)	252 (95.45)	114 (79.17)	Reference	Reference
	Positive	42 (10.29)	12 (4.55)	30 (20.83)	1.81 (0.80–4.09)	0.156
NAG	Negative	354 (86.76)	239 (90.53)	115 (79.86)	Reference	Reference
	Positive	54 (13.24)	25 (9.47)	29 (20.14)	1.47 (0.75–2.86)	0.263
GUS	Negative	320 (78.43)	218 (82.58)	102 (70.83)	Reference	Reference
	Positive	88 (21.57)	46 (17.42)	42 (29.17)	1.08 (0.60–1.97)	0.795

**Notes.**

H_2_O_2_ Hydrogen peroxide LEU Leukocyte esterase SNA sialidase NAG N-acetyl glucosidase GUS *β*-glucuronidase *P*-value probability value *CI* confidence interval *OR* odds ratio

## Discussion

UU, as a common reproductive tract pathogen, has become a critical threat to women’s health (Noh et al., 2019; Cicinelli et al., 2008). The factors contributing to UU infection in women were explored. The proportion of UU-positive participants who were cleanliness-positive, H_2_O_2_-positive, LEU-positive, SNA-positive, NAG-positive, or GUS-positive populations was higher than that of UU-negative populations. Cleanliness and H_2_O_2_ were found to be risk factors for UU infection in women. Consequently, to reduce vaginal microecological abnormalities and the incidence of UU infection and other diseases in women, more education should be imparted on healthy living for women.

UUs are pathogenic microorganisms that attach to various types of cells, such as epithelial and germ cells, and primarily colonize the mucosal surfaces of the genitourinary tract in adults. UU infections not only lead to reproductive tract inflammation but also to infertility and adverse outcomes of pregnancy, such as preterm premature rupture of the membrane (Li et al., 2010). This study found that a higher percentage of UU-positive participants had lower vaginal cleanliness than the UU-negative participants. No study has directly demonstrated the association between UU and vaginal cleanliness. However, cervical cancer and bacterial vaginosis are associated with abnormal vaginal cleanliness in patients (Zhang et al., 2023; Shen et al., 2024). Differences in the degree of vaginal cleanliness lead to differences in the abundance of vaginal microbial composition. Moreover, abnormalities in vaginal microbiota can cause diseases such as vaginitis (Liao et al., 2022). Thus, the vaginal microbiome plays a critical role in women’s health. UU-positive participants had a higher percentage of vaginal H_2_O_2_ positivity compared to the UU-negative participants. Although H_2_O_2_ is produced by probiotics such as lactobacilli, it can be used to assess vaginal health status, and abnormally normal levels of H_2_O_2_ may be associated with immune cytokines in vaginal secretions (Jacobsson et al., 2009; Hassanein et al., 2012; Noh et al., 2019; Mei & Li, 2022; Cai et al., 2022). Vaginal microecology, a complex system, is mediated by multiple factors, including normal anatomy, microflora, endocrine changes, and local immune responses (Ye & Qi, 2023). This is in accordance with the findings of our study that found a higher percentage of UU-positive participants with positive vaginal LEU, SNA, NAG, and GUS. Autoimmune status and viral infections were significantly associated with vaginal microbiota involved in the regulation of the immune system of the female lower genital tract, suggesting that the vaginal microflora dysbiosis may be a risk factor affecting persistent infections in the female genital tract (Pino et al., 2021). Therefore, vaginal cleanliness must be maintained, and vaginal microecology must be stable for women’s health.

An interdependence and mutual constraint exist between vaginal microecological-related factors, forming a dynamic balance system (Buggio et al., 2019). An imbalance in the vaginal microecosystem can be recognized when there is an alteration in indicators such as the number, species, dominant bacteria, pathogenic microorganisms, pH, and Lactobacillus function in the vagina (Wang et al., 2022; Ge et al., 2023). The analysis was performed between vaginal microecology and UU susceptibility. This study found that cleanliness was an independent risk factor for UU infection in patients. Similar to this study’s findings, several studies have identified the association of vaginal cleanliness with female reproductive diseases, including polycystic ovary syndrome and postpartum pelvic floor dysfunction (Hong et al., 2020; Cheng et al., 2022). The route of UU infection in the female genital tract is primarily through the vagina; therefore, extensive vaginal cleansing is critical. A low vaginal cleanliness grade indicates that a woman has a large number of Lactobacillus and epithelial cells in her vagina. Still, fewer leukocytes, and the vaginal microecology is in a healthy state. In a healthy female vagina, Lactobacillus produces H_2_O_2_, which protects the vagina. Garza et al.’s study found that the percentage of patients with UU infection who were positive for Lactobacillus in the vagina was lower than the percentage of Lactobacillus positivity in the healthy control population. Combined with our findings that high vaginal cleanliness is a risk factor for UU infection in patients, we hypothesize that Lactobacillus in the vagina and the Lactobacillus product, H_2_O_2_, may be essential factors in reducing UU infection in women. In the future, this study will further explore the mechanisms by which vaginal cleanliness level affects UU infection in women. In this study, H_2_O_2_ (*OR* = 7.24, 95% *CI* [4.19–12.51], *P* <  0.001), another indicator of vaginal microecology, was found to be an independent risk factor for UU infection in patients. Consistent with the above results, Li et al. found that abnormal levels of H_2_O_2_ could lead to cervical intraepithelial neoplasia and cervical squamous cell carcinoma (Zheng et al., 2019a; Zheng et al., 2019b). Stoyancheva found that H_2_O_2_-producing Lactobacillus has a vital role in protecting the vaginal microecological balance (Stoyancheva, Danova & Boudakov, 2006). Women who were negative for Lactobacillus vaginalis were more likely to be infected with Mycoplasma hominis, UU et al. than those who were positive for Lactobacillus in the vagina (Hillier et al., 1993). Vaginal H_2_O_2_ is mainly derived from Lactobacillus, so we hypothesized that H_2_O_2_ may be detrimental to UU infection in female patients. In the future, we will also explore the mechanism by which vaginal H_2_O_2_ reduces UU infection in women. Therefore, maintenance of vaginal microecological stability is critical to improve women’s health.

In addition, LEU, SNA, NAG, and GUS were found to be risk factors for UU infection in women in the univariate logistic analysis of this study. still, multivariate logistic analysis found that LEU, SNA, NAG, and GUS had no significant effect on UU infection in women. First, the results of univariate analysis are affected by other confounding factors, while multifactorial analysis corrects for the effects of other confounding factors, making the results more plausible. second, it is possible that whether LEU, SNA, NAG, or GUS is positive or not is indirectly related to UU infection in women. Finally, it may be that the sample size of this study is insufficient, and we will continue to collect samples for validation afterward. This study conducted a correlation analysis between SII and UU infection in women, but the results showed no significant correlation between SII and UU infection in women. However, it has been shown that there is an increase in polymorphonuclear leukocytes (PMNL) in the vagina of women with UU infection (Bender & Gundogdu, 2022). Afterward, we will test to examine further the relationship between other inflammatory markers and UU infection and analyze the role of inflammation in UU infection.

This study systematically investigated the association between vaginal microecology and UU infection. However, it has several limitations. First, the study population is primarily concentrated in northern China. Genetic differences, environmental factors, and cultural factors are all critical factors that may influence the results of the study, so in the future, we may also improve the generalizability of our findings through multicenter collaboration and cross-population comparisons for different populations to enhance the accuracy of our findings. Second, the exploration of potential mechanisms is insufficient. Vaginal cleanliness and H_2_O_2_ content are both associated with Lactobacillus in the vagina, so we hypothesized that Lactobacillus and Lactobacillus products such as H_2_O_2_ may be detrimental to UU infection in female patients. In the future, we will explore the mechanism of vaginal cleanliness and H_2_O_2_ content on UU infection in women for this speculation to further validate and improve our findings.

In conclusion, we found that UU-positive patients exhibited higher rates of cleanliness, H_2_O_2_, and other studied factors compared to UU negative patients. In addition, cleanliness and H_2_O_2_ were identified as independent risk factors for UU infection in women. Therefore, vaginal microecological examination should be strengthened in women to prevent UU infection caused by the abnormalities of the vaginal microecology.

##  Supplemental Information

10.7717/peerj.19783/supp-1 Supplemental Information 1 Raw data

## Additional Information and Declarations

### Data Availability

The following information was supplied regarding data availability: The raw are available in the Supplementary Files–Raw Data Required.

## References

[ref-1] Abou Chacra L, Fenollar F, Diop K (2022). Bacterial vaginosis: what do we currently know?. Frontiers in Cellular and Infection Microbiology.

[ref-2] Bender RA, Gundogdu C (2022). Cytological diagnosis of genital *ureaplasma urealyticum* and its importance in cervical inflammation. European Review for Medical and Pharmacological Sciences.

[ref-3] Buggio L, Somigliana E, Borghi A, Vercellini P (2019). Probiotics and vaginal microecology: fact or fancy?. BMC Womens Health.

[ref-4] Cai S, Wu Y, Zeng L, Ding Y (2022). Effects of vaginal microecology and immunity on the pregnancy outcome of cervical cerclage. BMC Womens Health.

[ref-5] Cheng C, Guo B, Li R, Wu W, Mi C, Li X (2022). Correlation between postpartum pelvic floor dysfunction and vaginal microecological imbalance in late pregnancy. Zhong Nan Da Xue Xue Bao Yi Xue Ban.

[ref-6] Cicinelli E, De Ziegler D, Nicoletti R, Colafiglio G, Saliani N, Resta L, Rizzi D, De Vito D (2008). Chronic endometritis: correlation among hysteroscopic, histologic, and bacteriologic findings in a prospective trial with 2,190 consecutive office hysteroscopies. Fertility and Sterility.

[ref-7] Doh K, Barton PT, Korneeva I, Perni SC, Bongiovanni AM, Tuttle SL, Skupski DW, Witkin SS (2004). Differential vaginal expression of interleukin-1 system cytokines in the presence of *Mycoplasma hominis* and *Ureaplasma urealyticum* in pregnant women. Infectious Diseases in Obstetrics and Gynecology.

[ref-8] Fan Z, Han D, Fan X, Zeng Y, Zhao L (2024). Analysis of the correlation between cervical HPV infection, cervical lesions and vaginal microecology. Frontiers in Cellular and Infection Microbiology.

[ref-9] Feng D, Zhang F, Cai J, Zhang Y, Yan H, Yang Y, Zhong H, Ye H (2024). Functional testing is a complementary tool for the diagnosis of vaginitis. BMC Womens Health.

[ref-10] Garza J, Gandhi K, Choi S, Sanchez A, Ventolini G (2021). Cytokine profiles and *Lactobacillus* species presence in pre-menopausal subjects with genital Mycoplasma genitalium or Ureaplasma urealyticum colonization. Womens Health.

[ref-11] Ge YM, Lu JC, Xu YH, Tang SS, Zhi SS, Liang YJ (2023). Correlations of joint detection of 22 vaginal microbes with routine examination results of vaginal secretions and assisted reproductive outcomes. Diagnostic Microbiology and Infectious Disease.

[ref-12] Hassanein SM, El-Farrash RA, Hafez HM, Hassanin OM, Abd El Rahman NA (2012). Cord blood interleukin-6 and neonatal morbidities among preterm infants with PCR-positive *Ureaplasma urealyticum*. Journal of Maternal-Fetal and Neonatal Medicine.

[ref-13] Hernández-Rodríguez C, Romero-González R, Albani-Campanario M, Figueroa-Damián R, Meraz-Cruz N, Hernández-Guerrero C (2011). Vaginal microbiota of healthy pregnant Mexican women is constituted by four Lactobacillus species and several vaginosis-associated bacteria. Infectious Diseases in Obstetrics and Gynecology.

[ref-14] Hillier SL, Krohn MA, Rabe LK, Klebanoff SJ, Eschenbach DA (1993). The normal vaginal flora, H_2_O_2_-producing lactobacilli, and bacterial vaginosis in pregnant women. Clinical Infectious Diseases.

[ref-15] Hong X, Qin P, Huang K, Ding X, Ma J, Xuan Y, Zhu X, Peng D, Wang B (2020). Association between polycystic ovary syndrome and the vaginal microbiome: a case-control study. Clinical Endocrinology.

[ref-16] Ilhan ZE, Łaniewski P, Thomas N, Roe DJ, Chase DM, Herbst-Kralovetz MM (2019). Deciphering the complex interplay between microbiota, HPV, inflammation and cancer through cervicovaginal metabolic profiling. EBioMedicine.

[ref-17] Jacobsson B, Aaltonen R, Rantakokko-Jalava K, Morken NH, Alanen A (2009). Quantification of *Ureaplasma urealyticum* DNA in the amniotic fluid from patients in PTL and pPROM and its relation to inflammatory cytokine levels. Acta Obstetricia et Gynecologica Scandinavica.

[ref-18] Li J, Hou Y, Zhao Y, Zhang ZM, Mao J (2010). Value of microbial gene 16SrRNA in the identification of antenatal infection. Zhongguo Dang Dai Er Ke Za Zhi.

[ref-19] Li L, Ding L, Gao T, Lyu Y, Wang M, Song L, Li X, Gao W, Han Y, Jia H, Wang J (2020). Association between vaginal micro-environment disorder and cervical intraepithelial neoplasia in a community based population in China. Journal of Cancer.

[ref-20] Liao Q, Zhang XF, Mi X, Jin F, Sun HM, Wang QX (2022). Influence of group B streptococcus and vaginal cleanliness on the vaginal microbiome of pregnant women. World Journal of Clinical Cases.

[ref-21] Liu P, Yu X, Wang J, Wang L, Ding Y, Liu J (2023). Establishment of pelvic inflammatory disease model induced by vaginal injection of *Ureaplasma urealyticum* liquids combined with fatigue and hunger. Animal Reproduction Science.

[ref-22] Mei Z, Li D (2022). The role of probiotics in vaginal health. Frontiers in Cellular and Infection Microbiology.

[ref-23] Moreno I, Simon C (2018). Deciphering the effect of reproductive tract microbiota on human reproduction. Reproductive Medicine and Biology.

[ref-24] Noh EJ, Kim DJ, Lee JY, Park JH, Kim JS, Han JW, Kim BC, Kim CJ, Lee SK (2019). Ureaplasma urealyticum infection contributes to the development of pelvic endometriosis through toll-like receptor 2. Frontiers in Immunology.

[ref-25] Pino A, Rapisarda AMC, Vitale SG, Cianci S, Caggia C, Randazzo CL, Cianci A (2021). A clinical pilot study on the effect of the probiotic Lacticaseibacillus rhamnosus TOM 22.8 strain in women with vaginal dysbiosis. Scientific Reports.

[ref-26] Plummer EL, Vodstrcil LA, Bodiyabadu K, Murray GL, Doyle M, Latimer RL, Fairley CK, Payne M, Chow EPF, Garland SM, Bradshaw CS (2021). Are *mycoplasma hominis*, Ureaplasma urealyticum and *ureaplasma parvum* associated with specific genital symptoms and clinical signs in nonpregnant women?. Clinical Infectious Diseases.

[ref-27] Rumyantseva T, Khayrullina G, Guschin A, Donders G (2018). Prevalence of ureaplasma spp. and mycoplasma hominis in healthy women and patients with flora alterations. Diagnostic Microbiology and Infectious Disease.

[ref-28] Shen L, Zhang W, Yuan Y, Zhu W, Shang A (2022). Vaginal microecological characteristics of women in different physiological and pathological period. Frontiers in Cellular and Infection Microbiology.

[ref-29] Shen Y, Zhu Q, Ren Y, Zhu Y, Xiang S, Xu J (2024). Analysis of the spectrum of common pathogens and the resistance of mycoplasma in sialidase-positive bacterial vaginosis. Clinical Laboratory.

[ref-30] Stoyancheva GD, Danova ST, Boudakov IY (2006). Molecular identification of vaginal *lactobacilli* isolated from Bulgarian women. Antonie Van Leeuwenhoek.

[ref-31] Wang L, He L, Chen J, Wei S, Xu H, Luo M (2022). HPV and vaginal microecological disorders in infertile women: a cross-sectional study in the Chinese population. Journal of Virology.

[ref-32] Wang S, Wan Y, Zhang W (2024). The clinical value of systemic immune inflammation index (SII) in predicting the severity of hospitalized children with mycoplasma pneumoniae pneumonia: a retrospective study. International Journal of General Medicine.

[ref-33] Wei W, Xie LZ, Xia Q, Fu Y, Liu FY, Ding DN, Han FJ (2022). The role of vaginal microecology in the cervical cancer. Journal of Obstetrics and Gynaecology Research.

[ref-34] Ye J, Qi X (2023). Vaginal microecology and its role in human papillomavirus infection and human papillomavirus associated cervical lesions. Acta Pathologica, Microbiologica, et Immunologica Scandinavica.

[ref-35] Yoshida T, Maeda S, Deguchi T, Ishiko H (2022). Phylogeny-based rapid identification of *mycoplasmas* and *ureaplasmas* from urethritis patients. Journal of Clinical Microbiology.

[ref-36] Zhang Z, Yang Y, Zhang L, Wu Y, Jia P, Ma Q, Wang D (2023). Relationship between cervicovaginal microecological changes and HPV16/18 infection and cervical cancer in women of childbearing age. Annals of Clinical & Laboratory Science.

[ref-37] Zheng JJ, Song JH, Yu CX, Wang F, Wang PC, Meng JW (2019b). Difference in vaginal microecology, local immunity and HPV infection among childbearing-age women with different degrees of cervical lesions in Inner Mongolia. BMC Womens Health.

[ref-38] Zheng N, Guo R, Yao Y, Jin M, Cheng Y, Ling Z (2019a). Lactobacillus iners is associated with vaginal dysbiosis in healthy pregnant women: a preliminary study. BioMed Research International.

